# The Impact of Bacteriocenoses on Sperm Vitality, Immunological and Oxidative Characteristics of Ram Ejaculates: Does the Breed Play a Role?

**DOI:** 10.3390/ani12010054

**Published:** 2021-12-28

**Authors:** Eva Tvrdá, Miroslava Kačániová, Andrej Baláži, Jaromír Vašíček, Jakub Vozaf, Rastislav Jurčík, Michal Ďuračka, Jana Žiarovská, Ján Kováč, Peter Chrenek

**Affiliations:** 1 Faculty of Biotechnology and Food Sciences, Slovak University of Agriculture, Tr. A. Hlinku 2, 94976 Nitra, Slovakia; evina.tvrda@gmail.com (E.T.); jaromir.vasicek@nppc.sk (J.V.); jakubvozaf@gmail.com (J.V.); michaelduracka@gmail.com (M.Ď.); jan.johnny.kovac@gmail.com (J.K.); 2 Institute of Horticulture, Faculty of Horticulture and Landscape Engineering, Slovak University of Agriculture, Tr. A. Hlinku 2, 94976 Nitra, Slovakia; kacaniova.miroslava@gmail.com; 3 Department of Bioenergetics, Food Analysis and Microbiology, Institute of Food Technology and Nutrition, University of Rzeszow, Cwiklinskiej 1, 35-601 Rzeszow, Poland; 4 NPPC, Research Institute for Animal Production Nitra, Hlohovecka 2, 95141 Luzianky, Slovakia; andrej.balazi@nppc.sk (A.B.); rastislav.jurcik@nppc.sk (R.J.); 5 Faculty of Agrobiology and Food Resources, Slovak University of Agriculture, Tr. A. Hlinku 2, 94976 Nitra, Slovakia; jana.ziarovska@uniag.sk

**Keywords:** bacteria, bacteriospermia, rams, native Wallachian, improved Wallachian, Slovak dairy, spermatozoa

## Abstract

**Simple Summary:**

Ram semen frequently presents with bacterial contamination, which may affect the resulting sperm vitality and fertilization ability. Since semen quality is of paramount importance for the successful artificial insemination of ewes, we focused on the description of bacterial profiles of ejaculates collected from three Slovak sheep breeds. Furthermore, we strived to unravel the effect of bacteriospermia on the immunological characteristics and oxidative profile of semen, both of which seem to play a role in bacteria-inflicted damage to male gametes.

**Abstract:**

Bacterial contamination of semen is an often overlooked, yet important, factor contributing to decreased sperm vitality. Understanding the impact of bacterial presence on sperm structural integrity and functional activity may assist the development of effective strategies to prevent, or manage, bacteriospermia in the breeding practice. The aim of this study was to describe the bacterial profiles of ram semen (*n* = 35), and we also focused on the associations between bacteriospermia, sperm structure, and function, as well as oxidative and inflammatory characteristics of semen. For a better insight, the samples were divided into three groups, according to the breeds used in the study: native Wallachian (NW), improved Wallachian (IW), and Slovak dairy (SD) breeds. The results showed a significantly lower motility and membrane integrity in the NW group in comparison to the IW and SD groups, which was accompanied by a significantly higher concentration of leukocytes, increased reactive oxygen species (ROS) generation, and subsequent oxidative insults to the sperm lipids and proteins. Accordingly, the NW group presented with the highest bacterial load, in which *Staphylococcus* and *Escherichia* were the predominant representatives. The Pearson correlation analysis uncovered positive relationships amongst the bacterial load and leukocytospermia (r = 0.613), the extent of lipid peroxidation (r = 0.598), protein oxidation (r = 0.514), and DNA fragmentation (r = 0.638). Furthermore, positive correlations were found between the bacterial load and pro-inflammatory molecules, such as the C-reactive protein (r = 0.592), interleukin 1 (r = 0.709), and interleukin 6 (r = 0.474), indicating a possible involvement of the immune response in the process of bacteriospermia. Overall, our data indicate that ram semen quality may be equally affected by the bacterial load and diversity. Furthermore, we can assume that the presence of bacteria in ejaculates triggers inflammatory processes, causes ROS overproduction, and, thereby, contributes to alterations in the sperm structure, while at the same time compromising the fertilization ability of male gametes.

## 1. Introduction

Modern reproductive technologies based on artificial insemination (AI) have become an indispensable pillar of livestock production. This technique, alongside other breeding strategies, such as induction and/or synchronization of estrus and ovulation, has significantly contributed to the remarkable progress in sheep breeding programs [1]. In comparison to natural breeding, AI is more effective in adding genetic value to livestock, by spreading the use of males with valuable traits, while reducing the risks of a potential horizontal or vertical transmission of sexual diseases [2]. At the same time, sperm cryopreservation helps to preserve the genetic material from historically or locally valuable breeds [3]. Nonetheless, the success of any reproductive technology by and large depends on the initial quality of the semen sample used for the respective procedure.

As reviewed by Petrovic et al. [4], numerous endogenous or exogenous factors may affect the quality of ram semen. Despite the great effort invested into securing the proper nutrition, welfare, and health status of the animal, there are several factors that are often overlooked; of which bacteriospermia has become an issue that needs to be taken more seriously. Bacteria present in ram ejaculates may stem from the urogenital system or preputial fluids, as well as from wool, skin, urine, feces, or respiratory secretions. Furthermore, contaminated feed and/or water, bedding, or poor hygiene standards in the breeding facility or the collection process may equally contribute to bacterial contamination of semen [5].

Previous studies on human and animal ejaculates have indicated that bacteriospermia may lead to sperm agglutination [6,7] and subsequent sub-standard sperm motility [8,9,10]. Furthermore, a greater proportion of spermatozoa with alterations to the membranous structures and morphological defects has been found in ejaculates contaminated with bacteria [9,10,11,12]. At the same time, it has been reported that bacteriospermia may induce an imbalance in the seminal oxidative balance, by accelerating reactive oxygen species (ROS) overproduction [13], an excessive release of pro-inflammatory cytokines, and subsequent damage to the sperm structures crucial for the male gamete to reach the oocyte and successfully accomplish fertilization [10,14]. Ultimately, bacteriospermia may be responsible for reduced shelf life of extended or cryopreserved semen samples [15]. Besides, uropathogenic bacteria may be easily transmitted to ewes and cause secondary infections in the female reproductive system, accompanied by lower conception rates and litter size [4,5].

To avoid such complications, the current European Council Directive no. 90/429/EEC enforces the supplementation of antibiotics to each semen sample used for semen processing, AI, or cryopreservation. Nevertheless, continuous use of antibiotics strengthens the occurrence, spread, and persistence of multidrug-resistant bacteria [16]. What is worse, resistance has been reported among several isolates collected from ram semen against antibiotics that are now frequently used in livestock production [15,17]. As such, the importance of understanding the bacterial profiles of ejaculates has an ever-increasing importance, in order to establish proper management or prevention of bacteriospermia in the future.

In comparison to routine microbiological analysis, involving a subjective description of morphological, biochemical, physiological (phenotype), and/or genetic characteristics (genotype) of the microorganism, our experimental strategy was based on matrix-assisted laser desorption/ionization time-of-flight mass spectrometry (MALDI-TOF MS), which has been successfully employed to study bacteriocenoses in bovine [10], stallion [18], turkey [9], rabbit [19], and boar semen [20]. Furthermore, rather than just focus on a global description on the bacterial profiles in ram semen and their implications on the resulting semen quality, we chose to adopt a comparative approach, by studying semen samples from three breeds (native Wallachian, improved Wallachian, and Slovak dairy), which have been classified among valuable genetic resources in Slovakia [21].

This study was designed to characterize the bacterial profiles of ram semen and to assess their possible impact on sperm structural and functional parameters. Moreover, we investigated these interactions in the broader context of the immunological and oxidative processes occurring in ejaculates during bacteriospermia.

## 2. Materials and Methods

### 2.1. Animal Keeping and Feeding

Clinically healthy and sexually mature rams of the native Wallachian (*n* = 3), improved Wallachian (*n* = 3), and Slovak dairy sheep (*n* = 3) breeds aged 2.5–5 years were used for the purposes of this study. The animals were housed under external conditions in individual stalls at a breeding facility (NPPC, Research Institute for Animal Production Nitra, Lužianky, Slovakia). They were fed with hay, mashed oats, and commercially available BAK feeding mixture (0.75–1.2 kg daily per ram; BAK producer: PPD Prašice, Jacovce, Slovakia) that consisted of organic wheat, organic barley, organic corn, and vitamin-mineral premix. Mineral salt and water were available ad libitum.

### 2.2. Semen Collection

Semen samples (11 samples from the native Wallachian breed; 10 samples from the improved Wallachian breed; 14 samples from the Slovak dairy sheep breed) were collected twice a week by electroejaculation (EE) within the autumn season (November–December 2020). Before the electroejaculation procedure, the rectum was cleaned of feces. If necessary, rams were anesthetized with xylazine (0.2 mg/kg Xylariem 2% a.u.v., Riemser Arzneimittel GmbH, Greifswald, Germany). A three-electrode probe for rams and boars with a diameter of 2.54 cm and length of approximately 16 cm attached to a power source allowing voltage and amperage control was used (Minitube Electroejaculator, Holzheim, Germany). The EE regime (automatic mode, type of curve 2: the power output was linearly increased from 0.5 V to 7 V) consisted of a consecutive series of 2 s pulses of similar voltage, each separated by 2 s breaks. The initial voltage was set to 0.5 V, which was then increased in each series until reaching a maximum of 7 V. Upon reaching 7 V, the impulses remained at this level until the ejaculation was complete. After collection, semen was transported to the laboratory in a thermal vessel at 30 °C.

Each native specimen was diluted at 1:40 in PBS (phosphate buffered saline; Sigma-Aldrich, St. Louis, MO, USA) and assessed for spermatozoa motion, occurrence of leukocytes, reactive oxygen species (ROS) generation, mitochondrial activity, DNA fragmentation, membrane, and acrosome integrity.

An aliquot of each semen sample was centrifuged (2300× *g*, 5 min, 20 °C), to collect the cell fraction and the seminal plasma separately. Aliquots of seminal plasma were kept at −80 °C, and subsequently subjected to an assessment of the total antioxidant status (TAS), interleukins 1 (IL-1), 2 (IL-2), 6 (IL-6), 8 (IL-8), 12 (IL-12), and C-reactive protein (CRP).

The sperm fraction was treated with RIPA buffer enriched with a protease inhibitor (Sigma-Aldrich, St. Louis, MO, USA) and lysed with an ultrasonic homogenizer (28 kHz) for 45 s. The resulting mixture was subsequently centrifuged (2300× *g*, 10 min, 4 °C) and purified. The obtained cell lysates were kept at −80 °C for the analysis of oxidative damage to the sperm proteins and lipids.

**Figure 1:**
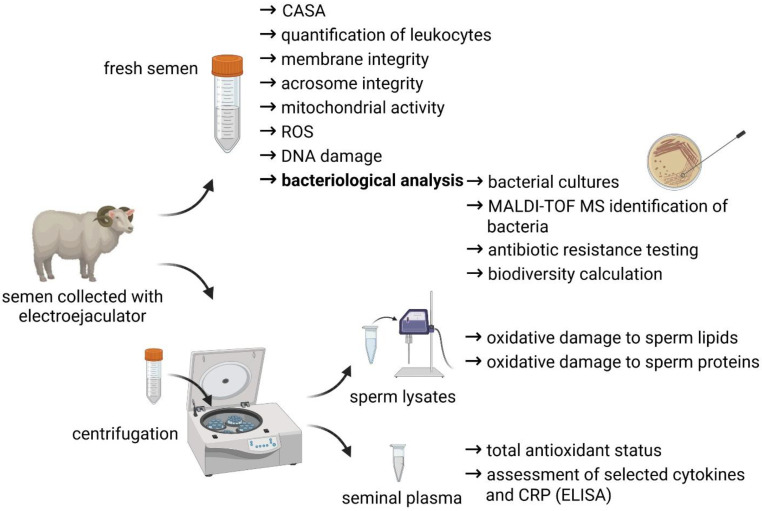
Experimental outline of the study.

A short overview of the experimental outline is provided by Figure 1.

### 2.3. Computer-Assisted Sperm Analysis

Ram sperm count, concentration, and motility were determined using computer-assisted sperm analysis (CASA; Version 14.0 TOX IVOS II.; Hamilton–Thorne Biosciences, Beverly, MA, USA). The CASA system was set according to the following cut-off values: frames acquired, 30; frame rate, 60 Hz; minimum contrast, 60; minimum static contrast, 15; cell size, 5 pixels; minimum cell size, 5 pixels; cell intensity, 55; static head size, 0.72–8.82; static head intensity, 0.14–1.84; and static elongation, 0–47. The IDENT dye served to stain the cells (Hamilton-Thorne Biosciences, Beverly, MA, USA), which were then analyzed under fluorescent illumination. A Makler counting chamber (Sefi Medical Instruments, Haifa, Israel) was used to load the specimen into the CASA system, and at least 300 sperm cells were evaluated per run. The count was determined in billions of spermatozoa, the concentration was expressed as 10^6^ spermatozoa/mL, while the motility was defined as the percentage of spermatozoa moving faster than 5 μm/s.

### 2.4. Quantification of Leukocytes

Leukocyte concentration was evaluated with the Endtz test. Twenty µL of diluted ejaculates (1:100) were treated with 40 µL of working solution comprised of 96% ethanol (Centralchem, Bratislava, Slovakia), benzidine (Sigma-Aldrich, St. Louis, MO, USA), sterile water, and 3% hydrogen peroxide (H_2_O_2_; Sigma-Aldrich, St. Louis, MO, USA), and subsequently incubated (dark conditions, 5 min, 20 °C). Stained round cells were quantified under a bright-field microscope (×1000; Nikon ECLIPSE E100, Tokyo, Japan). The results are expressed as ×10^6^ leukocytes/mL [10].

### 2.5. Membrane Integrity

To assess the sperm membrane integrity, 1 × 10^6^ cells were stained with 10 μL CFDA (carboxyfluorescein diacetate; Sigma-Aldrich, St. Louis, MO, USA; 0.75 mg/mL in DMSO), 10 μL DAPI (4′,6-diamidino-2-phenylindole; Sigma-Aldrich, St. Louis, MO, USA; 1 μmol/L in PBS) and incubated at 37 °C in the dark for 15 min. Afterwards, each sample was centrifuged (150× *g*, 5 min, 20 °C) and washed with 100 µL PBS twice. Finally, the samples were resuspended in 100 µL PBS, and a minimum of 300 cells were evaluated using an epifluorescence microscope with a ×40 magnification (Leica Microsystems, Wetzlar, Germany). CFDA-positive cells were classified as membrane-intact (%).

### 2.6. Acrosome Integrity

For the integrity of the acrosomal structures, 1 × 10^6^ cells were stained with 100 μL PNA (peanut agglutinin, FITC conjugate; Sigma-Aldrich, St. Louis, MO, USA; 10 μmol/L in PBS) and 10 μL DAPI. Following staining and incubation at 37 °C, in the dark for 15 min, the samples were analyzed with an epifluorescence microscope (×40 magnification). A minimum of 300 cells were counted, and PNA-negative spermatozoa were classified as acrosome-intact (%).

### 2.7. Mitochondrial Activity

Mitochondrial activity expressed through the mitochondrial membrane potential (ΔΨm) was evaluated with a JC-1 Mitochondrial Membrane Potential Assay kit (Cayman Chemical, Ann Arbor, MI, USA). The JC-1 fluorescent dye (5.5′,6.6′-tetrachloro-1,1′,3,3′-tetraethylbenzimidazolylcarbocyanine iodide) was dissolved in PBS to constitute the working solution (5 μmol/L JC-1), 5 μL of which was mixed with 1 × 10^6^ cells adjusted to 100 μL and incubated at 37 °C for 30 min. Subsequently, each sample was centrifuged (150× *g*, 5 min, 20 °C) and washed twice with a JC-1 washing buffer. Finally, the specimens were transferred to a dark 96-well plate and analyzed using a GloMax-Multi + fluorometer (Promega, Madison, WI, USA). The resulting ΔΨm is expressed as the green/red ratio (JC-1 complexes/JC-1 monomers) [19].

### 2.8. Sperm DNA Damage

Sperm DNA fragmentation index (%) was evaluated with a Halomax commercial kit (Halotech DNA, Madrid, Spain). A small aliquot (20 μL) of each ejaculate was mixed with agarose. Subsequently, 10 μL of the mixture was placed on microscopic slides pre-coated with agarose, covered with coverslips, and left at 4 °C for 5 min. Following solidification, the slides were treated with a lysis buffer (5 min), distilled water (5 min), ethanol (70% and 100%; 2 min each), and air-dried. Each slide was stained with SYBR Green (2 μg/mL in DMSO; Sigma-Aldrich, St. Louis, MO, USA) and Vectashield (Vector Laboratories, Burlingame, CA, USA), and a minimum of 300 sperm cells were evaluated with an epifluorescence microscope (×40 magnification) [19].

### 2.9. Reactive Oxygen Species (ROS) Generation

The extent of ROS production was quantified with a chemiluminescent approach. The tested specimens consisted of 10 μL 5 mmol/L luminol (Sigma-Aldrich, St. Louis, MO, USA) and 400 μL of diluted samples. Negative controls comprised 400 μL PBS. Positive controls contained 400 μL of the sample and 50 μL H_2_O_2_ (30%; Sigma-Aldrich, St. Louis, MO, USA). The resulting luminescence was monitored in fifteen cycles of 1 min on 48-well microplates with a Glomax Multi+ luminometer (Promega, Madison, WI, USA). The results are expressed in relative light units (RLU)/s/10^6^ sperm [10].

### 2.10. Total Antioxidant Status

Total antioxidant status of the ram seminal plasma was assessed with a chemiluminescent assay involving horseradish peroxidase (Sigma-Aldrich; St. Louis, MO, USA), 4-iodophenol (Sigma-Aldrich; St. Louis, MO, USA), luminol, and H_2_O_2_. Trolox (5–100 μmol/L; 6-hydroxy-2,5,7,8-tetramethylchroman-2-carboxylic acid; Sigma-Aldrich; St. Louis, MO, USA) served for the construction of the standard curve. The luminescent signal was monitored on 96-well plates during 10 consecutive cycles of 1 min with a Glomax Multi+ luminometer. The results are expressed as μmol Trolox Eq./g protein [10].

### 2.11. Enzyme-Linked Immunosorbent Assay (ELISA)

Selected cytokines (IL-1, IL-2, IL-6, IL-8, and IL-12) and CRP were determined using commercially available ELISA kits, designed for samples of sheep origin (MyBioSource Inc., San Diego, CA, USA). All assays followed a double-sandwich ELISA protocol and were executed with the help of a Glomax plate spectrophotometer (Promega, Madison, WI, USA) at 450 nm.

### 2.12. Oxidative Damage to Sperm Lipids

A thiobarbituric acid-reactive substances (TBARS) assay was employed to assess the production of malondialdehyde (MDA), considered to be a primary byproduct of lipid peroxidation (LPO). The sperm lysates (100 μL) were pre-treated with 100 μL 5% SDS (sodium dodecyl sulfate; Sigma-Aldrich, St. Louis, MO, USA) and 4 mL 0.53% thiobarbituric acid (Sigma-Aldrich, St. Louis, MO, USA), previously dissolved in 20% acetic acid (pH 3.5; Centralchem, Slovakia). Subsequently, the samples were exposed to heat (100 °C) in a water bath for 60 min. To cease the ongoing reaction, the samples were cooled down for 10 min and subsequently centrifuged (1300× *g*, 10 min, 4 °C). The supernatants were pipetted into a 96-well plate, and the absorbances were measured with a Glomax plate spectrophotometer at 540 nm. MDA levels were calculated with the help of a standardization curve constructed from pre-prepared MDA standards. The extent of LPO is expressed in µmol/L MDA/g protein [22].

### 2.13. Oxidative Damage to Sperm Proteins

To evaluate protein oxidation, the amount of protein carbonyls (PC) in the sperm lysates was quantified with a modified DNPH (dinitrophenylhydrazine) assay [23]. Each sample was diluted with distilled water to contain 1 mg protein/1 mL, and subsequently treated with 1 mL of trichloroacetic acid (TCA; Sigma-Aldrich, St. Louis, MO, USA) and cooled down for 10 min at 4 °C. Each sample was then centrifuged (805× *g*, 10 min, 4 °C), and the resulting pellet was incubated (60 min, 37 °C) with 1 mL DNPH (Sigma-Aldrich, St. Louis, MO, USA). Afterwards, 1 mL TCA was added once more, the samples were cooled down and centrifuged again (805× *g*, 5 min, 4 °C). The pellet was washed 3 times with 500 µL ethyl acetate/ethanol solution, 50/50 mix (Sigma-Aldrich, St. Louis, MO, USA). Finally, the resulting pellet was resuspended in 1 mL 6 M guanidine hydrochloride (Sigma-Aldrich, St. Louis, MO, USA). The quantification of protein carbonyls was performed at 360 nm using a UV-VIS spectrophotometer (Cary Systems, Santa Clara, CA, USA). Oxidative damage to the proteins is expressed in nmol PC/mg protein [22].

### 2.14. Data Normalization

To normalize the data obtained from the assessments of the seminal plasma and sperm lysates, it was fundamental to quantify the amount of proteins in each specimen. Aliquots of seminal plasma and sperm lysates were processed with a commercial Total Protein kit (DiaSys, Holzheim, Germany), and the total concentration of proteins in the samples was determined using a Monza photometric analyzer (Randox Laboratories, Crumlin, UK) at 540 nm.

### 2.15. Bacterial Cultures

The first step in the identification of the bacterial species in ram semen consisted in an inoculation of 100 µL of each specimen being inoculated onto a selection of sterile agars (blood agar base no. 2; Gassner agar, NutriSelect^®^ basic; soybean casein digest agar; Merck, Darmstadt, Germany). The cultures were then incubated under aerobic conditions at 36 ± 2 °C for 24 h. Subsequently, bacterial colonies were counted and transferred to a fresh tryptone soya agar to obtain pure cultures, which were incubated again under aerobic conditions at 37 ± 1 °C for 24 h [10].

### 2.16. Bacteriological Identification

The isolated and purified bacterial cultures were identified using a MALDI-TOF Biotyper mass spectrometry (Brucker Daltonics, Bremen, Germany).

A small amount of a purified culture was re-suspended in 300 μL of distilled water. Subsequently, 900 μL of 99.8% ethanol (Centralchem, Bratislava, Slovakia) was administered and the mixture was centrifuged (920× *g*, 2 min, 20 °C). The resulting pellet was allowed to dry freely and then mixed vigorously with 30 μL of 70% formic acid (Sigma-Aldrich, St. Louis, MO, USA) and 30 μL of acetonitrile (Sigma-Aldrich, St. Louis, MO, USA). The samples were subsequently centrifuged at 1096× *g* at 20 °C for 2 min. Then, 1 μL of the supernatant was placed on the MALDI identification plate, allowed to dry freely and subsequently covered with a working solution of MALDI matrix, composed of acetonitrile, ultrapure water, trifluoroacetic acid, and cinnamic acid (Sigma-Aldrich, St. Louis, MO, USA). Identification of the isolates was carried out with a Microflex LT instrument and the flexControl software version 3.4. The spectra obtained by MALDI-TOF were linked with the MALDI Biotyper Bruker Taxonomy database (Bruker Daltonics, Bremen, Germany) [10].

### 2.17. Antibiotic Resistance Testing

Bacterial isolates identified in semen samples collected from three sheep breeds were furthermore tested for antibiotic resistance. An antimicrobial susceptibility test was executed with the disc diffusion method against (10 mg) amikacin, chloramphenicol, ciprofloxacin, gentamicin, imipenem, lefloxacin, meropenem, norfloxacin, tigecycline, and tobramycin, following the protocol published by Kačániová et al. [24].

### 2.18. Biodiversity Calculation

One-way ANOVA was used to determine the differences among analyzed samples of three breeds of rams using the Astasta calculator. Heatmaps of bacterial abundance among the analyzed breeds were prepared in MS Excel. The overall number of species obtained from the pre-established groups of ram semen samples was defined as species richness. Standard α-, β-, and γ-diversity parameters were calculated with the BPMSG diversity calculator. Shannon alpha entropy was calculated following the formula: $H_{\alpha }=-w_{1}\overset{N}{\underset{i=1}{\sum }}p_{i1}\ln\;p_{i1}+-w_{2}\overset{N}{\underset{i=1}{\sum }}p_{i2}\ln\;p_{i2}+\cdots +w_{K}\overset{N}{\underset{1}{\sum }}p_{iK}\ln\;p_{iK}$ with *p_ij_* being the relative abundance (frequency, priority, share) of class *i* and sample *j* and *w_i_* statistical weights of samples; $\overset{K}{\underset{i=1}{\sum }}w_{i}=1$. The Berger–Parker Index was obtained following the formula d = max(pi) to describe real unbalanced groups different among the breeds.

### 2.19. Statistical Analysis

The GraphPad Prism program (version 8.4.4 for Mac; GraphPad Software Incorporated, La Jolla, CA, USA) was used for the statistical analysis. The results are expressed as mean ± standard deviation (S.D.). Collected data were processed with the Pearson correlation analysis. The interpretation of the data was dependent on the Pearson correlation coefficient (r): ±0.111–±0.333: weak correlation; ±0.334–±0.666: moderate correlation; ±0.667–±0.999: strong correlation. To understand the deeper causality of the obtained data, the samples were distributed according to the breed, into a native Wallachian group (NW; *n* = 11), improved Wallachian group (IW; *n* = 10), and Slovak dairy group (SD; *n* = 14). Differences between the established groups were analyzed by one-way ANOVA and Tukey multiple comparison test. Statistical significance for both operations was set at: * *p* < 0.05; ** *p* < 0.01; *** *p* < 0.001; **** *p* < 0.0001.

## 3. Results

### 3.1. Identification of Bacteria

**Figure 2:**
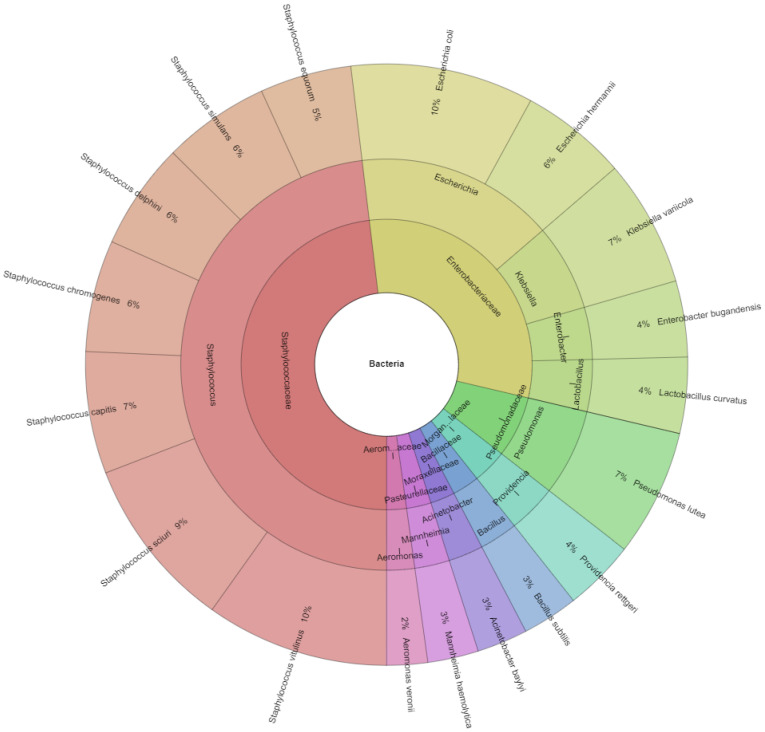
Krona chart of the bacteria represented by the MALDI-TOF MS Biotyper, recovered from ram semen (outermost ring: species, middle ring: genus, innermost ring: family).

Using MALDI TOF mass spectrometry, eight families, 11 genera, and 18 bacterial species were uncovered in the ram semen samples (Figure 2): *Acinetobacter baylyi* (*A. baylyi*), *Aeromonas veronii* (*A. veronii*), *Bacillus subtilis* (*B. subtilis*), *Enterobacter bugandensi* (*E. bugandensi*), *Escherichia coli* (*E. coli*), *Escherichia hermannii* (*E. hermannii*), *Klebsiella variicola* (*K. variicola*), *Lactobacillus curvatus* (*L. curvatus*), *Mannheimia haemolytica* (*M. haemolytica*), *Providencia rettgeri* (*P. rettgeri*), *Pseudomonas lutea* (*P. lutea*), *Staphylococcus delphini* (*S. delphini*), *Staphylococcus capitis* (*S. capitis*), *Staphylococcus chromogenes* (*S. chromogenes*), *Staphylococcus equorum* (*S. equorum*), *Staphylococcus sciuri* (*S. sciuri*), *Staphylococcus simulans* (*S. simulans*), and *Staphylococcus vitulinus* (*S. vitulinus*).

Overall, 366 bacterial isolates were identified in ram semen with a score higher than 2.00. The predominant genera present in ram ejaculates were *Staphylococcus* (49%) and *Escherichia* (16%). Gram-positive bacteria were more frequent (56%) in comparison to Gram-negative bacteria (44%).

### 3.2. Comparative Analysis of the Bacterial Profiles of Semen

The highest number of bacterial colonies (Table 1) was observed in the NW group. Significant differences (*p* < 0.05) were detected in comparison to the IW, as well as the SD, group.

Ejaculates distributed in the NW group were contaminated with species representing primarily the *Staphylococcus* and *Escherichia* genera. Furthermore, *M. haemolytica*, *B. subtilis*, and *K. variicola* were identified in the NW group. While *E. coli*, *S. vitulinus*, *S. sciuri*, and *L. curvatus* were detected in the IW group; *E. coli*, *S. chromogenes*, *A. veronii*, *S. vitulinus*, *S. capitis*, *S. equorum*, *P. rettgeri*, *A. baylyi*, and *P. lutea* were isolated from semen samples belonging to the SD group.

### 3.3. Biodiversity Assessment

**Figure 3:**
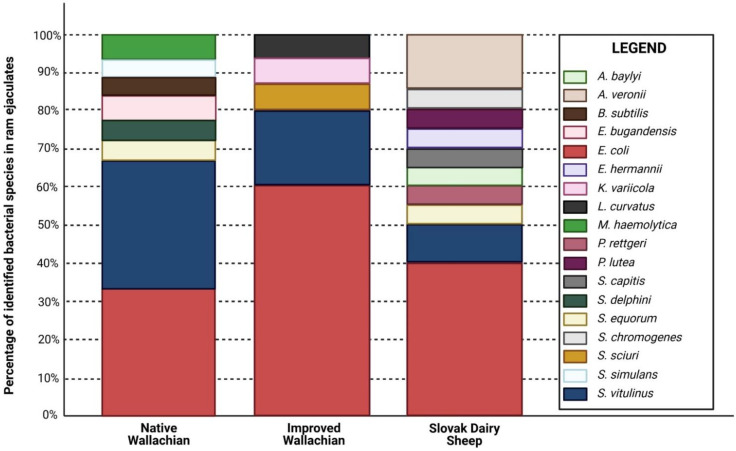
Heatmap of bacterial species identified in the ejaculates of individual ram breeds.

A total of 18 different bacterial species were identified in the analyzed samples. The most abundant species in all three groups were *E. coli* and *S. vitulinus* (Figure 3). The bacterial profile of the SD group was the most diverse, when compared to the other two groups.

According to the biodiversity calculation (Table 2), the highest bacterial richness was found in the SD group. The Berger–Parker Index values were low in all groups, which indicates a relatively small domination of individual bacterial species in the analyzed samples. Moreover, similar values of the Shannon index amongst the pre-established groups may have been affected by the low abundance and number of bacteria that were identified in the semen samples.

### 3.4. Antimicrobial Resistance

All bacterial isolates were subjected to antimicrobial resistance assessment (Table 3) against amikacin, chloramphenicol, ciprofloxacin, gentamicin, imipenem, lefloxacin, meropenem, norfloxacin, tigecycline, and tobramycin. The resulting inhibition zones were evaluated according to the EUCAST (European Committee on Antimicrobial Susceptibility Testing) guidelines. It was revealed that only one *S. equorum* and one *S. vitulinus* strain were resistant to ciprofloxacin, otherwise all tested isolates were sensitive to the antibiotics used for the test.

### 3.5. Correlation Analysis

The Pearson correlation analysis (Table 4) uncovered an array of negative effects of bacterial contamination on ram semen quality. We recorded a strong negative correlation between the motion parameters and the bacterial load (*p* < 0.001). At the same time, strong positive associations (*p* < 0.001) were observed among the presence of leukocytes, ROS production, and the bacterial load.

Meanwhile, ROS amounts were in a negative correlation with the motion characteristics (*p* < 0.001) and sperm cell structures vulnerable to oxidative insults, including the membrane (*p* < 0.001), acrosome (*p* < 0.01), and mitochondria (*p* < 0.01).

While the integrity of the plasma membrane was strongly positively associated with the motility and progressive motility (*p* < 0.001), the parameter was in a strong negative correlation (*p* < 0.001) with the extent of the lipid peroxidation, as well as the quantity of bacteria present in the ejaculates (*p* < 0.001). Interestingly, acrosome integrity was not significantly affected by the bacterial load.

The mitochondrial activity was in a strong positive association (*p* < 0.001) with the sperm motility characteristics, while exhibiting a significant negative relationship with the bacterial load (*p* < 0.001), ROS amount (*p* < 0.01), and subsequent oxidative damage to the sperm proteins (*p* < 0.01) and lipids (*p* < 0.001).

A strong positive correlation was recorded between the bacterial load and the extent of protein oxidation (*p* < 0.01) and MDA (*p* < 0.001), while being negatively associated with the antioxidant capacity of the seminal plasma (*p* < 0.01). Moreover, we observed a significant positive correlation between the number of bacteria present in the ejaculates and DNA fragmentation (*p* < 0.001). Furthermore, DNA damage was significantly associated with the presence of leukocytes and ROS production (*p* < 0.001).

All pro-inflammatory markers of the seminal plasma (interleukins and CRP) exhibited a strong negative association with sperm motility (*p* < 0.001), while being positively associated with CFU (*p* < 0.001 in case of IL-1 and CRP; *p* < 0.01 with respect to IL-2, IL-6, and IL-12; *p* < 0.05 with regards to IL-8).

At the same time, we observed that particularly IL-1 (*p* < 0.001), CRP, and IL-12 (*p* < 0.01) were negatively associated with the antioxidant status of the seminal plasma, while exhibiting a positive association with the amounts of ROS (*p* < 0.001) and MDA (*p* < 0.001). All inflammatory molecules had a significant negative correlation with the sperm concentration and motility, as well as progressive motility (*p* < 0.001).

For a better understanding of the causality of the collected data, the samples were divided into three groups, based on the breed of the rams used in the study: Native Wallachian group (NW; *n* = 11), Improved Wallachian group (IW; *n* = 10), and Slovak dairy groups (SD; *n* = 14).

### 3.6. Comparative Analysis of Standard Semen Parameters

**Figure 4:**
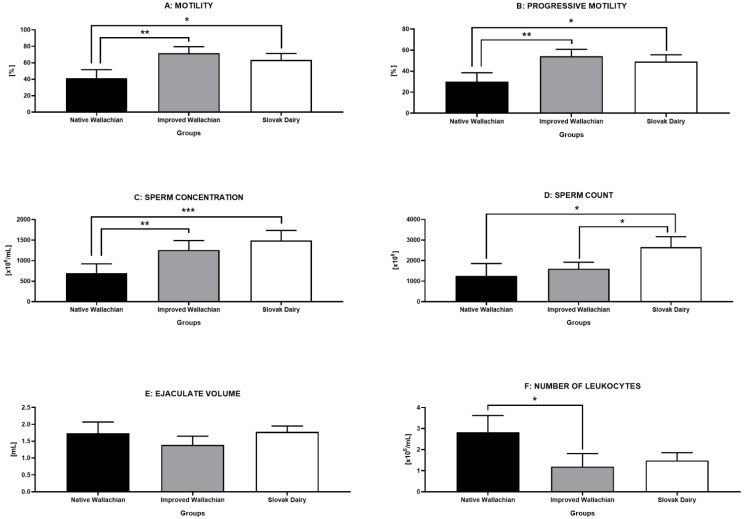
Comparative analysis of standard semen characteristics among the pre-established groups of ram semen samples. * *p* < 0.05; ** *p* < 0.01; *** *p* < 0.001.

The sample distribution analysis revealed that both motility and progressive motility were significantly decreased in the NW group, when compared to the IW (*p* < 0.01) and the SD group (*p* < 0.05) (Figure 4A,B). Similarly, the sperm concentration (Figure 4C), as well as the total sperm count (Figure 4D), were significantly lower in the NW group in contrast to the IW and SD group. Even though the ejaculate volume did not differ significantly among the groups (Figure 4E), the highest concentration of leukocytes was detected in the NW group (*p* < 0.05 in comparison to IW; Figure 4F).

### 3.7. Comparative Analysis of Sperm Structural Integrity Markers

**Figure 5:**
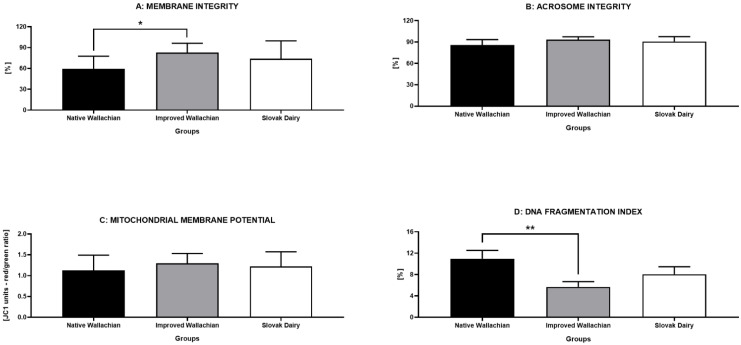
Comparative analysis of the sperm structural integrity parameters among the pre-established groups of ram semen samples. * *p* < 0.05; ** *p* < 0.01.

Evaluation of the sperm structural integrity revealed a significantly lower proportion of spermatozoa with intact membranes in the NW group, in comparison to the IW group (*p* < 0.05) (Figure 5A). While no differences among the groups were detected with respect to the acrosome integrity (Figure 5B) or the mitochondrial membrane potential (Figure 5C), a significantly higher percentage of spermatozoa with fragmented DNA were observed in the NW group, particularly in comparison to the IW group (*p* < 0.01) (Figure 5D).

### 3.8. Comparative Analysis of the Seminal Oxidative Profile

**Figure 6:**
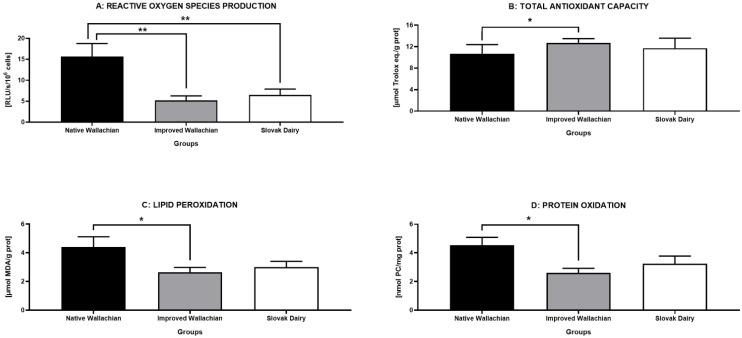
Comparative analysis of the oxidative profile markers among the pre-established groups of ram semen samples. * *p* < 0.05; ** *p* < 0.01.

With respect to the assessment of the oxidative profile we recorded a significantly higher ROS amount in the NW group in comparison to the IW and SD groups (*p* < 0.01) (Figure 6A). An imbalance in the oxidative profile of semen specimens collected from native Wallachian rams was evident from a significantly decreased antioxidant capacity of the seminal plasma (*p* < 0.06 in comparison to IW) (Figure 6B). Accordingly, significantly increased oxidative damage to the sperm lipids (Figure 6C) and proteins (Figure 6D) was observed in the ejaculates belonging to the NW group, particularly in comparison to the IW group (*p* < 0.06).

### 3.9. Comparative Analysis of Pro-Inflammatory Markers

**Figure 7:**
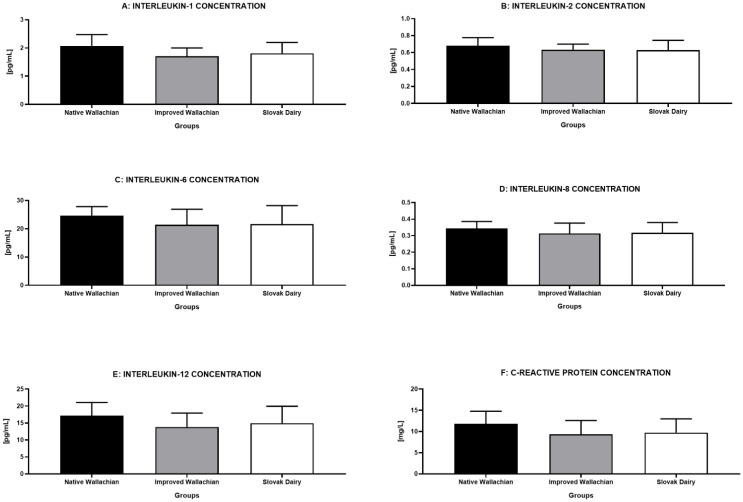
Comparative analysis of the pro-inflammatory markers among the pre-established groups of ram semen samples.

The highest levels of all pro-inflammatory factors were recorded in the NW group (Figure 7A–F), although no significant differences were detected among the pre-established groups.

## 4. Discussion

While numerous factors such as nutrition, age, heat stress, and seasonality are traditionally acknowledged to affect sperm quality in rams, bacteriospermia has increasingly attracted attention as an often overlooked, yet important, aspect that should be taken seriously for further semen handling and its respective use in reproductive technologies. As such, this study focused on the identification and description of the bacterial profiles of ram semen in the broader context of their impact on standard semen parameters, sperm structural integrity, and oxidative and immunological markers of the ejaculates.

The results from the bacteriological analysis revealed that ram semen contains a relatively high load of microorganisms. As previously reported by Anel-Lopez et al. [17], the bacterial load of cryopreserved ram semen was determined to be around 1.80 log_10_ CFU/mL, which is lower in comparison to our data (5.68–7.03 log_10_ CFU/mL); nevertheless, this discrepancy may be explained by the fact that the semen samples in the above-mentioned study had been extended for the freezing process before the microbiological examination was performed. On the other hand, Yániz et al. [15] stated that native ram ejaculates can be infested with up to 10 log_10_ CFU/mL. What is more, Azawi and Ismaeel [25] observed that the bacterial load may be strongly affected by the season of ram semen collection, with the highest values reaching up to 138.30 CFU/mL. According to the authors, the average bacterial counts for the winter period oscillated around 62.50 CFU/mL, which corresponds to the values obtained in the period of sample collection in this study (November–December).

Most of the samples tested positive for *Staphylococcus*, *Escherichia*, and *Pseudomonas*, which is consistent with Anel-Lopez et al. [17]. Similarly to Yániz et al. [15], we also identified *Acinetobacter*, *Enterobacter*, *Klebsiella*, and *Morganella*; however, *Proteus*, *Salmonella*, *Serratia*, and *Streptococcus* were absent in our samples. On the other hand, *Bacillus* and *Lactobacillus* were found in this study.

The most uniform bacterial representation was observed in semen samples collected from the native Wallachian rams, in which *S. vitulinus* was the most predominant species, however without any of the negative effects previously reported in spermatozoa. On the other hand, *E. coli* was detected relatively abundantly in all groups, which has been recognized as an uropathogen capable of significantly affecting sperm vitality and fertilization potential in vivo, as well as in vitro [26].

The impact of bacteriospermia on sperm concentration has been repeatedly emphasized in studies on animal and human semen. It was previously reported that oligozoospermia, or even azoospermia, was observed in subjects with acute epididymitis [5], although we can speculate that this phenomenon may be observed in clinically healthy subjects without any obvious signs of an infection, and agreeing with Moretti et al. [8], who observed a significant bacterial presence in semen samples collected in apparently healthy and fertile males. As such, we may hypothesize that if the animals indeed were suffering from a ‘hidden’ urogenital infection, it was possibly recent, before the first symptoms could manifest themselves.

Our data reveal that the sperm motion characteristics declined correspondingly with increasing bacterial load. Moreover, the sperm motility behavior was affected by the concentration as well as composition of the bacteria present in the semen specimens. The mechanisms of action by which bacteria cause sperm motility inhibition are different. Some bacteria, including *Escherichia* and *Klebsiella*, adhere to the sperm surface and/or receptors on the sperm flagellum, leading to motility suppression [27,28]. Other bacterial species, such as representatives of the *Staphylococcus* genus, may cause permanent sperm agglutination, through the secretion of agglutination or immobilization factors [29]. Furthermore, bacterial metabolites such as lipopolysaccharide, hemolysin, and peptidoglycan fragments may cause the flagellum to degenerate, tear off, or break, which has a negative effect on the ability of spermatozoa to reach the oocyte [30,31,32]. The exact mechanism by which such damage occurs is not yet fully understood, nevertheless previous reports indicated a significant role of membrane disintegration, promoted by ROS overproduction, either directly by the bacteria or indirectly by damaged spermatozoa [33]. Furthermore, it was revealed that the presence of bacteria may significantly decrease the availability of calcium and magnesium, which are crucial for proper mitochondrial function, leading to a lower production of the ATP crucial for sperm movement [10].

It was reported in previous studies that the negative effects of bacteriospermia may often be mediated by the elevated amounts of leukocytes present in semen [13,34]. A positive association between the bacterial load and the leukocyte concentration observed in our study complements previous reports on cattle [10], human [13], and avian semen [9]. While phagocytosis is the most described role of seminal leukocytes [35], these may be equipped with other mechanisms to eliminate bacteria, such as ROS overproduction and the construction of extracellular traps (ETs). While the primary role of both events is to prevent the loss of sperm survival under undesirable conditions, several authors have indicated that the presence of ETs is accompanied by elevated ROS production, as well as an increased risk of physical capture of male reproductive cells, leading to a motility standstill [36,37]. Meanwhile, our results indicate that elevated concentrations of seminal leukocytes were positively associated with ROS overgeneration and a reduced sperm motility, which is why we can speculate that ETs may emerge in ram ejaculates in a similar manner.

The correlation analysis revealed strong inverse associations between interleukins, CRP, and most markers of sperm structural integrity and functional activity. In agreement with our results, previous studies on animal semen [9,10] emphasized the complex network of negative interactions between the molecules of the immune system and sperm vitality. The release of cytokines leads to the activation of eosinophils, neutrophils, and macrophages, which is accompanied by an elevated oxygen uptake and a subsequent oxidative outburst [38]. As such, cytokines alone increase the sperm susceptibility to oxidative insults, while additional ROS released by the immune cells lead to further deterioration of the male reproductive cell. Other studies indicated that the inflammatory molecules released during the immune response may contribute to sperm DNA damage [39]. This hypothesis was strengthened by our data, according to which an elevated oxidative tension in semen was strongly associated with increased levels of pro-inflammatory cytokines, lipid peroxidation of the sperm membrane lipids, and DNA fragmentation, as a result of the loss of membrane integrity and the direct interplay between ROS and the sperm chromatin.

Our data indicate that seminal ROS had a negative impact on all sperm quality markers. Furthermore, the highest ROS production was recorded in samples containing the highest bacterial load and the richest bacterial diversity. As such, we may speculate that the primary mechanism of bacteria-inflicted damage to male gametes may lie in an imbalance of the seminal prooxidant–antioxidant milieu. This phenomenon has previously been observed in human [40], bovine [10], and turkey semen samples [9]; thus, we may agree with the assumption that the bacterial load could be directly responsible for elevated ROS levels in semen. Spermatozoa, as well as aerobic bacteria, are inherently predetermined to produce ROS as a byproduct of their aerobic metabolism, while both present with their own antioxidant defense mechanisms, to counteract elevated oxidative tension [33,41]. The onset of an infection is proportional to the ability of bacteria to handle the immune reaction of the host organism [42], accompanied by a massive ROS release by the phagocytic cells. Nevertheless, ROS levels considered lethal for bacteria will have negative implications for sperm survival as well. It is well established that the primary location for ROS-insults are the polyunsaturated fatty acids, located in the sperm membranes. High ROS levels may then manifest themselves in a significant rise of MDA, which may lead to high proportions of spermatozoa with distorted membranes [43]. Sperm membrane disintegration may be also caused by bacterial toxins, the accumulation of which on the sperm surface leads to the formation of small pores, facilitating an uncontrolled Na^+^ influx, leading to mitochondrial swelling and rupture, which is accompanied by intracellular release of ROS [44,45].

One of the most frequent consequences of bacteriospermia reported in previous studies, is a significant increase in sperm DNA fragmentation [9,10,46,47]. While an elevated sperm DNA fragmentation index may be directly associated with the physical presence of bacteria in semen, the extent of DNA damage may be affected by a variety of factors, such as the bacterial load or growth rate. An elevated proportion of male gametes with a damaged DNA molecule in groups with a higher bacterial presence and diversity might stem from a previous higher frequency of oxidative insults to the DNA molecule [40,48]. This hypothesis is also reinforced by strong correlations between sperm DNA damage, leukocytospermia, ROS levels, and lipid peroxidation, as well as concentrations of pro-inflammatory molecules in our study. At the same time, the presence of bacterial endotoxins has also been linked to the onset of apoptosis or necrosis, which manifest themselves with the loss of DNA stability [47]. Similarly to our data, bovine, turkey, and human semen samples infested with *Staphylococcus* and *Escherichia* presented with high levels of DNA damage, accompanied by the loss of motility and membrane fluidity [9,10,47].

Based on our results, we may hypothesize that the loss of semen quality in ram as a result of bacteriospermia, may be driven by oxidative mechanisms, rather than the immune response, since the comparative analysis did not reveal significant differences in the levels of pro-inflammatory molecules among the pre-established groups. This may support the above-mentioned speculation that if, indeed, the animals had suffered from a urogenital infection, it must have been a recent one. As such, we may propose the following sequence of events accompanying bacterial contamination of ram ejaculates: the presence of bacteria in semen leads to ROS overproduction as a primary line of defense from seminal leukocytes, as well as by the bacteria themselves. The prime target for excessive ROS are the lipid components of the sperm membranes, which will subsequently undergo lipid peroxidation accompanied by a rise of MDA levels [40,49]. Sperm membranes compromised by oxidative insults will lose fluidity and permeability, and such membrane destabilization will result in protein inactivation, mitochondrial rupture, and the loss of the internal environment of the sperm cell, which will be accompanied by the loss of motion behavior [40,50]. In summary, we may hypothesize that ROS overgeneration accompanied by insufficient antioxidant defense mechanisms may be the primary reason for the sperm dysfunction during bacteriospermia.

To our knowledge, this study represents the first attempt to describe and compare the markers for sperm structural integrity and functional activity, immunological, and oxidative parameters in three historically important Slovak sheep breeds. While a handful of studies have compared standard semen parameters in such breeds, with no significant differences found [51,52,53,54], our results reveal that the lowest sperm vitality was found in ejaculates collected from the native Wallachian breed. At the same time, samples from native Wallachian rams presented with a higher vulnerability to bacterial contamination and oxidative stress. Since all animals came from one breeding facility, were similar of age, and kept under identical conditions, these factors may be excluded in this study. The animals were sexually mature and had been undergoing regular semen collection with pre-established hygiene standards. Nevertheless, only a routine health assessment was performed before the electroejaculation, which may be considered as an important limitation of this study. While the animals did not show any signs of distress, discomfort, or disease, the data obtained from the measurements of the proinflammatory molecules strongly suggest that an early or asymptomatic infection could have been ongoing. As such, we can hypothesize that the quantification of interleukins and CRP from the blood serum may have revealed an ongoing infection, and, thereby, this provides more soundness to the interpretation of the obtained data.

Bacterial contamination of animal ejaculates has created the prerequisite to use semen extenders that can provide an effective protection of male reproductive cells against the structural or functional damage inflicted by the presence of bacteria. As such, all media used for semen processing and storage must conform to European Directive 90/429/EEC and carry antibiotics to avoid or suppress bacterial overgrowth. Since bacterial resistance to antibiotics has become an indisputable phenomenon in animal breeding, the selection of the most suitable antibiotic supplement for semen extenders has become an important step in assuring the best sperm quality for subsequent AI procedures. According to our data, the highest sensitivity of the bacteria isolated from ram semen samples was observed in case of amikacin and gentamycin. As such, we can recommend these two antibiotics as supplements for the handling of ram ejaculates.

## 5. Conclusions

In conclusion, this study reveals that the bacterial load, as well as diversity, in ram ejaculates affects the architecture and functional manifestations of male gametes, as well as the oxidative and inflammatory properties of ram ejaculates. Generally speaking, the presence of bacteria induces a complex response, which results, particularly, in the loss of membrane and DNA integrity of the male gametes. Reactive oxygen species seem to play a critical role as messengers of the immune response, and directly impact sperm structures involved in the process of fertilization. Finally, our study emphasizes the importance of the bacteriological screening of semen samples used for reproductive technologies, as well as of future studies focused on the respective impacts of the recovered bacterial species on sperm performance.

## Figures and Tables

**Table 1:** Bacterial profiles of ram semen samples according to the breed.

Groups	Bacterial Species Identified in the Samples and Sample Positivity	Bacterial Colonies (log_10_ CFU/mL)
Native Wallachian (*n* = 11)	*S. vitulinus* (63%), *E. coli* (54%), *S. delphini* (9%), *S. simulans* (9%), *S. equorum* (9%), *E. bugandensis* (9%), *M. haemolytica* (9%), *B. subtilis* (9%), *E. hermannii* (9%), *K. variicola* (9%)	7.03 ± 0.53
Improved Wallachian (*n* = 10)	*S. vitulinus* (90%), *E. coli* (30%), *S. sciuri* (10%), *L. curvatus* (10%)	5.68 ± 0.78 *^NW^
Slovak Dairy (*n* = 14)	*S. chromogenes* (57%), *E. coli* (21%), *A. veronii* (21%), *S. vitulinus* (14%), *S. capitis* (7%), *S. equorum* (7%), *P. rettgeri* (7%), *A. baylyi* (7%), *P. lutea* (7%)	5.79 ± 0.63 *^NW^

* *p* < 0.05. ^NW^—in comparison to the NW group.

**Table 2:** Bacterial biodiversity characteristics of the pre-established groups of ram semen samples.

Quality Groups	NW	IW	SD
Average population size	2.375	3	2.2
Richness (R)	8	5	10
Berger–Parker (Dominance) Index	0.368	0.6	0.363
Shannon α-diversity	1.662	1.17	1.972

NW—native Wallachian; IW—improved Wallachian; SD—Slovak dairy.

**Table 3:** Resistance profiles of bacteria recovered from ram semen samples.

Bacterium	Isolate				
		IMP	MEM	GEN	TOB
*Acinetobacter baylyi*	1	S	S	S	S
		CIP	LEV		
*Aeromonas veronii*	1	S	S		
	2	S	S		
	3	S	S		
		TGC	C	CIP	TOB
*Bacillus subtilis*	1	ND	ND	ND	ND
		TOB	C	AMK	NOR
*Enterobacter bugandensis*	1	S	S	S	S
		TOB	C	AMK	NOR
*Escherichia coli*	1	S	S	S	S
	2	S	S	S	S
	3	S	S	S	S
	4	S	S	S	S
	5	S	S	S	S
	6	S	S	S	S
	7	S	S	S	S
	8	S	S	S	S
	9	S	S	S	S
	10	S	S	S	S
	11	S	S	S	S
	12	S	S	S	S
	13	S	S	S	S
	14	S	S	S	S
	15	S	S	S	S
	16	S	S	S	S
	17	S	S	S	S
	18	S	S	S	S
	19	S	S	S	S
	20	S	S	S	S
	21	S	S	S	S
	22	S	S	S	S
		TOB	C	AMK	NOR
*Escherichia hermannii*	1	S	S	S	S
		TGC	C	CIP	TOB
*Klebsiella variicola*	1	S	S	S	S
		TGC	C	CIP	TOB
*Lactobacillus curvatus*	1	ND	ND	ND	ND
		TGC	C	CIP	TOB
*Mannheimia haemolytica*	1	ND	ND	ND	ND
		TOB	C	AMK	NOR
*Providencia rettgeri*	1	S	S	S	S
		MEM	TOB	AMK	CIP
*Pseudomonas lutea*	1	S	S	S	S
		TGC	C	CIP	TOB
*Staphylococcus capitis*	1	S	S	S	S
		TGC	C	CIP	TOB
*Staphylococcus chromogenes*	1	S	S	S	S
	2	S	S	S	S
	3	S	S	S	S
		TGC	C	CIP	TOB
*Staphylococcus delphini*	1	S	S	S	S
		TGC	C	CIP	TOB
*Staphylococcus equorum*	1	S	S	R	S
	2	S	S	S	S
		TGC	C	CIP	TOB
*Staphylococcus sciuri*	1	S	S	S	S
		TGC	C	CIP	TOB
*Staphylococcus simulans*	1	S	S	S	S
		TGC	C	CIP	TOB
*Staphylococcus vitulinus*	1	S	S	S	S
	2	S	S	S	S
	3	S	S	S	S
	4	S	S	S	S
	5	S	S	S	S
	6	S	S	R	S
	7	S	S	S	S
	8	S	S	S	S
	9	S	S	S	S
	10	S	S	S	S
	11	S	S	S	S
	12	S	S	S	S
	13	S	S	S	S
	14	S	S	S	S
	15	S	S	S	S

TOB—Tobramycin, IMP—Imipenem, TGC—Tigecyklin, AMK—Amikacin, C—Chloramphenicol, NOR—Norfloxacin, LEV—Lefloxacin, GEN—Gentamycin, CIP—Ciprofloxacin, MEM—Meropenem, ND—not defined, S—sensitive, R—resistant.

**Table 4:** Associations between the bacterial load, ram semen quality, oxidative, and immunological markers (*n* = 35).

	MOT	PRO	CON	CNT	VOL	LEU	MI	AI	ΔΨm	DNA	ROS	TAC	LPO	PC	CRP	IL-1	IL-2	IL-6	IL-8	IL-12	CFU
MOT	1	0.987	0.736 ***	0.642 ***	0.130	−0.752 ***	0.798 ***	0.482 **	0.671 ***	−0.827 ***	−0.831 ***	0.607 ***	−0.767 ***	−0.690 ***	−0.787 ***	−0.822 ***	−0.654 ***	−0.614 ***	−0.588 ***	−0.770 ***	−0.795 ***
	PRO	1	0.779 ***	0.647 ***	0.112	−0.759 ***	0.810 ***	0.535 ***	0.678 ***	−0.817 ***	−0.819 ***	0.628 ***	−0.750 ***	−0.678 ***	−0.780 ***	−0.780 ***	−0.613 ***	−0.594 ***	−0.566 ***	−0.752 ***	−0.778 ***
		CON	1	0.766 *	−0.008	−0.544 ***	0.640 ***	0.551 ***	0.550 ***	−0.673 ***	−0.596 ***	0.549 ***	−0.627 ***	−0.542 ***	−0.654 ***	−0.596 ***	−0.543 ***	−0.628 ***	−0.521 ***	−0.649 ***	−0.602 ***
			CNT	1	0.473 **	−0.389 **	0.432 **	0.280	0.440 **	−0.491 **	−0.467 **	0.335 *	−0.474 **	−0.444 **	−0.524 ***	−0.518 ***	−0.468 **	−0.440 **	−0.350 *	−0.491 **	−0.607 ***
				VOL	1	0.027	−0.091	−0.282	−0.161	−0.004	0.113	−0.229	0.214	0.063	−0.109	−0.020	−0.064	−0.056	−0.032	−0.032	−0.178
					LEU	1	−0.741 ***	−0.626 ***	−0.643 ***	0.711 ***	0.695 ***	−0.690 ***	0.647 ***	0.533 ***	0.608 ***	0.584 ***	0.425 *	0.344 *	0.289	0.510 **	0.613 ***
						MI	1	0.766 ***	0.624 ***	−0.890 ***	−0.746 ***	0.831 ***	−0.790 ***	−0.697 ***	−0.819 ***	−0.814 ***	−0.644 ***	−0.578 ***	−0.529 **	−0.764 **	−0.614 ***
							AI	1	0.592 ***	−0.557 **	−0.554 **	0.780 ***	−0.525 **	−0.415 **	−0.467 *	−0.402 *	−0.287	−0.254	−0.152	−0.404 *	−0.282
								ΔΨm	1	−0.488 **	−0.579 **	0.636 ***	−0.611 ***	−0.518 **	−0.485 **	−0.514 **	−0.365 *	−0.236	−0.226	−0.495 **	−0.621 ***
									DNA	1	0.716 ***	−0.721 ***	0.774 ***	0.743 ***	0.827 ***	0.851 ***	0.756 ***	0.698 ***	0.665 ***	0.807 ***	0.638 ***
										ROS	1	−0.689 ***	0.869 ***	0.785 ***	0.704 ***	0.788 ***	0.642 ***	0.535 **	0.524 **	0.677 ***	0.613 ***
											TAC	1	−0.702 ***	−0.667 ***	−0.529 **	−0.584 **	−0.403 *	−0.356 *	−0.292	−0.533 ***	−0.506 **
												LPO	1	0.859 ***	0.791 ***	0.908 ***	0.785 ***	0.683 ***	0.656 ***	0.822 ***	0.598 ***
													PC	1	0.768 ***	0.800 ***	0.774 ***	0.709 ***	0.681 **	0.834 ***	0.514 **
														CRP	1	0.867 ***	0.881 ***	0.842 ***	0.807 ***	0.917 ***	0.592 ***
															IL-1	1	0.850 ***	0.739 ***	0.748 ***	0.896 ***	0.709 ***
																IL-2	1	0.885 ***	0.857 ***	0.900 ***	0.470 **
																	IL-6	1	0.914 ***	0.895 ***	0.474 **
																		IL-8	1	0.850 ***	0.403 *
																			IL-12	1	0.605 **
																				CFU	1

Data interpretation followed the value of the Pearson’s correlation coefficient: ±0.111–±0.333: weak correlation; ±0.334–±0.666: moderate correlation; ±0.667–±0.999: strong correlation. * *p* < 0.05; ** *p* < 0.01; *** *p* < 0.001. MOT: sperm motility (%); PRO: sperm progressive motility (%); CON: sperm concentration (million/mL); CNT: sperm count (billions); VOL: semen volume (mL); LEU: concentration of leukocytes (×10^6^/mL); MI: membrane integrity (%); AI: acrosome integrity (%); ΔΨm: mitochondrial membrane potential (JC-1 units); DNA: sperm DNA fragmentation (%);ROS: reactive oxygen species production (RLU/s/10^6^ cells); TAC: total antioxidant species (eq. µmol Trolox/g prot); MDA: malondialdehyde concentration (lipid peroxidation) (µmol MDA/g prot); PC: protein carbonyls content (protein oxidation) (nmol PC/mg prot); CRP: C-reactive protein (mg/g prot); IL: interleukins (pg/mg prot); CFU: colony-forming units (log CFU/mL).

## Data Availability

The data presented in this study are available on request from the corresponding author. Figure 1 and Figure 3 were created with BioRender.com (accessed on 17 December 2021).
